# Integration of scRNA-Seq and Bulk RNA-Seq to Analyse the Heterogeneity of Ovarian Cancer Immune Cells and Establish a Molecular Risk Model

**DOI:** 10.3389/fonc.2021.711020

**Published:** 2021-09-21

**Authors:** Leilei Liang, Jing Yu, Jian Li, Ning Li, Jing Liu, Lin Xiu, Jia Zeng, Tiantian Wang, Lingying Wu

**Affiliations:** Department of Gynecologic Oncology, National Cancer Center/National Clinical Research Center for Cancer/Cancer Hospital, Chinese Academy of Medical Sciences and Peking Union Medical College, Beijing, China

**Keywords:** ovarian cancer, scRNA-seq, myeloid cells, 2-gene signature, risk, prognosis

## Abstract

**Background:**

Considerable evidence suggests that the heterogeneity of ovarian cancer (OC) is a major cause of treatment failure. Single-cell RNA sequencing (scRNA-seq) is a powerful tool to analyse the heterogeneity of the tumour at the single-cell level, leading to a better understanding of cell function at the genetic and cellular levels.

**Methods:**

OC scRNA-seq data were extracted from the Gene Expression Omnibus (GEO) database and the FindCluster () package used for cell cluster analysis. The GSVA package was used for single-sample gene set enrichment analysis (ssGSEA) analysis to obtain a Hallmark gene set score and bulk RNA-seq data were used to analyse the key genes of OC-associated immune cell subsets. CIBERSORT was used to identify immune scores of cells and the “WGCNA” package for the weighted correlation network analysis (WGCNA). KEGG (Kyoto Encyclopedia of Genes and Genomes) and GO (Gene Ontology) analyses of subtype groups were performed by GSEA. Then, univariate Cox and lasso regression were performed to further establish a signature. Finally, qPCR and immunohistochemistry staining were used to evaluate the expression of signature genes in OC.

**Results:**

Two scRNA-seq (GSE154600 and GES158937) datasets were integrated to obtain 20 cell clusters. T cells or NK cells (cluster 5, 6, 7, 11), B cells (cluster 16, 19, 20) and myeloid cells (cluster 4, 9, 10) were clustered according to immune cell markers. The ssGSEA revealed that M1- and M2-like myeloid cell-related genes were significantly upregulated in P3 and P4 patients in the GSE154600 data. Immune cell analysis in TCGA-OC showed that a high abundance of M1-like tumour-associated macrophages (TAMS) predicts better survival. WGCNA, univariate Cox and lasso Cox regression established a two-gene signature (RiskScore=-0.059*CXCL13-0.034*IL26). Next, the TCGA-test and TCGA-OC were used to test the risk prediction ability of the signature, showing a good effect in the datasets. Moreover, the qPCR and immunohistochemistry staining revealed that the expression of CXCL13 and IL26 was reduced in OC tissues.

**Conclusion:**

A two-gene signature prognostic stratification system (CXCL13 and IL26) was developed based on the heterogeneity of OC immune cells to accurately evaluate the prognostic risk.

## Introduction

Ovarian cancer (OC) is a common gynaecologic malignancy with high mortality. The mainstay of treatment for ovarian cancer is a combination of surgery and chemotherapy, however, the 5-year survival rate for OC is approximately 47%, primarily due to a high recurrence rate and drug resistance (1). With its unique mechanism of action and relatively safe profile, immunotherapy has recently emerged as a promising modality for numerous malignancies, including OC. However, clinical studies showed that the anti-programmed cell death ligand-1/programmed cell death-1 (PD-L1/PD-1) axis in OC indicates an objective response rate (ORR) of only 10-15%, even if the CPS (Cyber Physical Systems) score is above 10, the remission rate is only 17.1%. The advances have demonstrated that OC with sufficient heterogeneity contributes to treatment failure and a poor prognosis (2).

Single-cell RNA sequencing (scRNA-Seq) uses optimised next-generation sequencing technologies to define the global gene expression profiles of single cells, facilitating dissection of the previously hidden heterogeneity in cell populations (3). In previous studies, scRNA-seq was used to characterise OC heterogeneity to develop novel therapeutic approaches based on the JAK/STAT-pathway inhibitor (4). Hu et al. used scRNA-seq to identify six subtypes of fallopian tube epithelium (FTE) cells in normal human fallopian tube tissues revealing intra-tumoural heterogeneity in serous ovarian cancer (SOC) and defined SOC subtypes that correlated with patient prognosis (5). Recently, researchers demonstrated the broad utility of scRNA-seq for discovering immunotherapy emerging standard of care for several cancer types because it could help the immune system to fight cancer cells (6). For example, scRNA-seq analyses were performed on the immune tumour microenvironment in colorectal cancer patients, providing evidence of the importance of Bhlhe40+ Th1-like CD4+ T cells in anti-tumour immunity and immunotherapy (7). Peng Junya et al. employed scRNA-seq in pancreatic cancer, identifying a subset of ductal cells with unique proliferative features that were associated with an inactivation state in tumour-infiltrating T cells, providing novel markers for the prediction of the antitumor immune response (8). Therefore, analysis of key genes based on the immune heterogeneity could provide potential immunotherapy targets and meaningful risk prediction for OC.

In this study, a series of tissue-specific clusters were constructed to predict immune cell compositions from two OC scRNA-seq (GSE154600 and GES158937) datasets in the Gene Expression Omnibus (GEO) database. Normalisation and variance stabilisation of single-cell RNA-seq data using regularised negative binomial regression was performed using SCTransform () and the FindCluster () package was used for immune cell clusters analysis. Bulk RNA-seq from the TCGA (The Cancer Genome Atlas) expression profile data was used to analyse the key genes in the OC-associated immune cell subsets. Next, we performed univariate Cox, lasso Cox regression and stepwise regression to establish a signature, with qPCR and immunohistochemistry performed to evaluate the expression of signature genes in OC. Finally, a two-gene signature prognostic stratification system (CXCL13 and IL26) was developed based on the heterogeneity of OC immune cells to identify potential immunotherapy targets and accurately evaluate the prognostic risk.

## Methods

### Data Download

OC scRNA-seq data GSE154600 including 5 high-grade SOC patients, 33538 genes and 52121 cells as well as GES158937 including 3 high-grade SOC patients, 36601 genes and 15202 cells were download from GEO databases (**Supplementary Table 1**). TCGA-OC bulk RNA-seq data including 378 ovarian cancer patients and 32484 genes were download from TCGA databases (**Supplementary Table 2**).

### scRNA-Seq Data Processing

The Seurat package SCTransform () function was used to pre-process and reduce the batch effect to integrate the two single-cell transcriptome datasets. The most changed 3000 genes were chosen by SelectIntegrationFeatures () (**Supplementary Table 3**) and the FindCluster () package used for immune cell cluster analysis with the resolution set to 0.15.

### ssGSEA

Single-sample GSEA (ssGSEA) analysis was performed using the GSVA package to obtain a hallmark gene set score and the Hallmark gene set was obtained from MSigDB. Spearman’s coefficient was used to evaluate the correlation between EMT, carcinogenesis and the p53 pathway.

### WGCNA

CIBERSORT was used to estimate the abundance of 22 immune cells in the TCGA-OC bulk RNA-seq data. The “WGCNA” package was used for the weighted correlation network analysis (WGCNA). β is the most important parameter in the analysis process, and β = 5 was used for subsequent analysis. For hub genes, the genes with module membership (MM) >0.5 and a Pearson correlation coefficient of 0.1 with overall survival (OS) were selected.

### NMF Algorithm to Identify Molecular Subtypes

First, gene expression data were extracted from the TCGA-OC database and randomly divided into a training group and test group. Then, the training data of NMF was collected, with the NMF method selecting the standard “brunet” for 10 iterations. The cluster number K was set at 2 to 10, and the average contour width of the common member matrix was determined by the R package “NMF” and the training samples were divided into two categories.

### Identification and Functional Analysis of Differentially Expressed Genes

The DEGs between group 1 and group 2 were calculated by the limma package, then screened with FDR <0.05 and |log2FC|> 2 to identify the differences. Furthermore, KEGG functional enrichment analysis was performed using the Clusterprofiler (V3.16.1) package.

### Support Vector Machine

The imvigor 210 cohort includes information on the immune infiltration type of 348 patients. An SVM model was constructed with the “e1071” package to predict the type of immune infiltration.

### Molecular Risk Model Construction

The coxph () function of the survival package was used to fit the Cox risk regression and a p-value<0.05 was considered as survival related. The least absolute shrinkage and selection operator (Lasso) method is a compression estimation that obtains a more refined model by constructing a penalty function, thereby compresses some coefficients and setting some coefficients to zero at the same time. Therefore, the advantage of subset shrinkage is retained. It is a biased estimation for processing data with multicollinearity that can realise the selection of variables while estimating parameters to better solve the problem of multicollinearity in regression analysis. We used the glmnet package to perform lasso Cox regression for analysis and 10-fold cross-validation for model construction.

### Specimen Collection

Ovarian tumour and normal tissues derived from surgically resected specimens were snap-frozen in liquid nitrogen and stored at -80°C until RNA extraction. No patients received chemotherapy, radiation therapy or received treatment before surgery. All patients signed informed consent forms provided by the Cancer Hospital, CAMS & PUMC. This study was approved by the Ethics Committee of the Cancer Institute (Hospital), CAMS & PUMC (17-099/1355).

### Total RNA Extraction and Quantitative Real-Time PCR

Total RNA extraction was performed using RNA-easy Isolation Reagent (No.RC112-01, Vazyme, China) from 10 ovarian tumour and 4 non-tumour tissues. Then, quantitative real-time PCR (qRT-PCR) was performed using the HiScript III 1st Strand cDNA Synthesis Kit (No.R312-01, Vazyme, China) and ChamQTM Universal SYBR^®^ qPCR Master Mix (No.Q712-02, Vazyme, China) according to the manufacturer’s instructions. The primer sequences were as follows: CXCL13 Forward Sequence 5’-3’: TATCCCTAGACGCTTCATTGATCG and Reverse Sequence 5’-3’: CCATTCAGCTTGAGGGTCCACA; IL26 Forward Sequence 5’-3’: GGAAGACGTTTTTGGTCAACTGC and Reverse Sequence 5’-3’: CTCTCTAGCTGATGAAGCACAGG; GAPDH Forward Sequence 5’-3’: GTCTCCTCTGACTTCAACAGCG and Reverse Sequence 5’-3’: ACCACCCTGTTGCTGTAGCCA. GAPDH served as an internal control.

### IHC Staining

An immunohistochemistry SP kit (No. SP-9000, ZSGB-BIO, China) was used for IHC, which was performed as previously described (9). Anti-CXCL13 (1:200) and anti-IL26 (1:200) were purchased from Abcam (ab272874 and ab254476). The magnification of the immunohistochemistry images was 20×.

### Statistical Analysis

All statistical analyses were performed using R software 3.5.3 and GraphPad Prism v. 8.01 (GraphPad Software, La Jolla, CA, USA). The Student’s t-test was used to compare values between the test and control groups and P-values < 0.05 were considered significant.

## Results

### Integration and Clustering of scRNA-Seq Data

**Figure 1:**
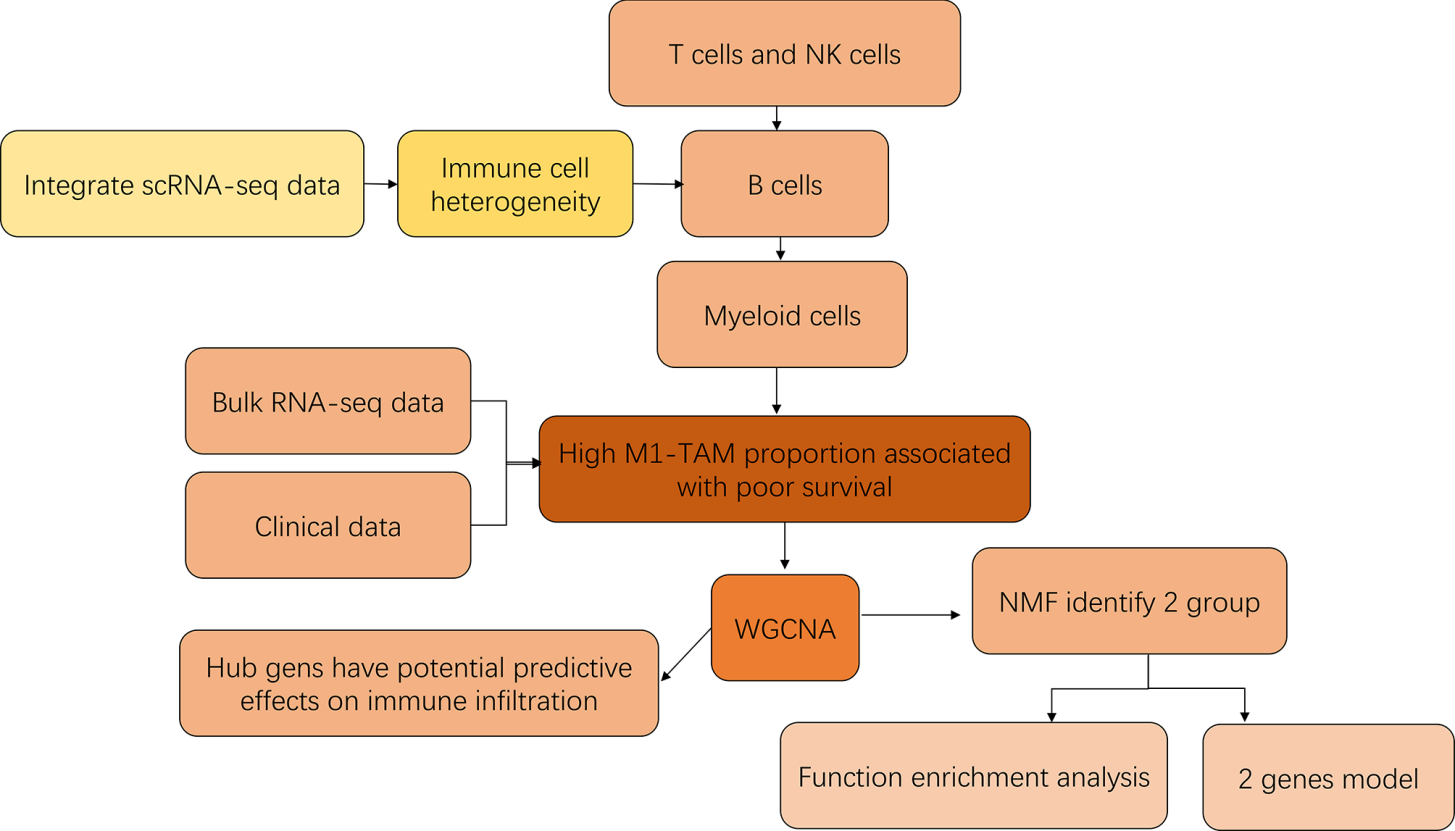
The technical road map.

**Figure 2:**
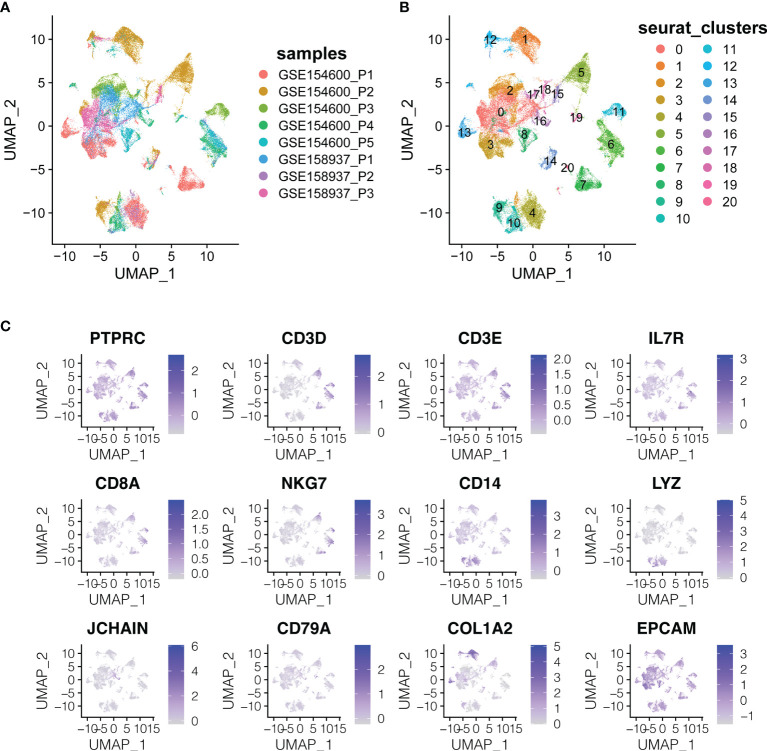
The dimension reduction of OC scRNA-seq. **(A)** Color depending on different patients. **(B)** Label colors according to separate clusters. **(C)** Expression of important marker genes.

Two scRNA-seq datasets (GSE154600 and GES158937) (**Table 1** and **Figure 1**) were used to characterise the OC heterogeneity in the GEO database. To integrate two single-cell transcriptome datasets, the Seurat package SCTransform () function was used to pre-process and reduce the batch effect. Uniform Manifold Approximation and Projection (UMAP) was used for non-linear dimension reduction (**Figure 2A**). The FindCluster () function was used to cluster cells, obtaining 20 clusters (**Figure 2B**).

**Table 1:** OV patient information (single-cell RNA-seq).

Study ID	Sample	Sample ID	Stage	Grade
GSE154600	GSE154600_P1	GSM4675273	stage: IV	G3
	GSE154600_P2	GSM4675274	stage: III	G3
	GSE154600_P3	GSM4675275	stage: IV	G3
	GSE154600_P4	GSM4675276	stage: IV	G3
	GSE154600_P5	GSM4675277	stage: IV	G3
GSE158937	GSE158937_P1	GSM4816045	NA	NA
	GSE158937_P2	GSM4816046	NA	NA
	GSE158937_P3	GSM4816047	NA	NA

T cells or NK cells (cluster 5, 6, 7, 11; markers: CD3D and CD3E), B cells (cluster 16, 19, 20; marker: CD79A) and myeloid cells (cluster 4, 9, 10; LYZ and CD14) were clustered according to immune cells markers (PTPRC is an immune cell marker; EPCAM is an epithelial cell marker; COL1A2 is a fibroblast marker; IL7R is the naive T cell marker; CD8A and NKG7 are CD8+ the T cell and NK cell markers) (**Figure 2C**).

### Immune Cell Analysis

**Figure 3:**
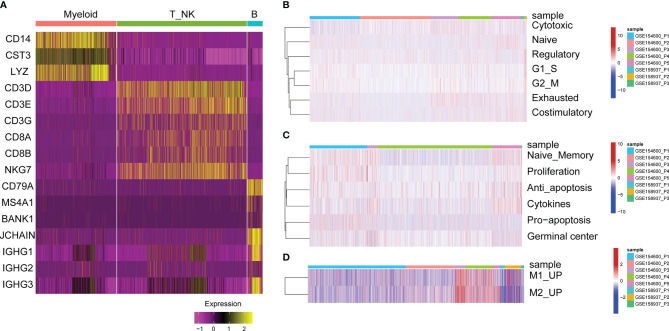
GSVA enrichment analysis of immune cells. **(A)** Heat map of significant marker gene expression in immune cells. **(B)** T cell characterization in OC. GSVA enrichment fractions of naive T cell, co-stimulatory T cells, Regulatory T cells, and exhausted T cells related gene sets. **(C)** B cell characterization in OC. GSVA enrichment fraction of naive B cells, proliferative, anti-apoptotic, pro-apoptotic, cytokine and germinal center related gene sets. **(D)** Characteristics of myeloid cells in OC. GSVA enrichment fraction of relating gene sets in M1 and M2-like myeloid cells.

Cluster analysis of T cells or NK cells, B cells and myeloid cells was based on immune cell markers (**Figure 3A**). First, we classified and identified T cells, then cluster analysis was performed based on the GSVA enrichment score of each sample of cells. According to the T cell functional status, such as regulatory, costimulatory, initial, cytotoxic, and exhaustive, the gene expression characteristics of naive T cells, costimulatory T cells, regulatory T cells, and exhausted T cells were identified (**Figure 3B**).

Second, the functional status of B cells was analysed, such as anti-apoptosis, naive memory, cytokines, proliferation and germinal centre gene expression characteristics (**Figure 3C**). For tumour-infiltrated myeloid cells, the activity of M2 and M1-like myeloid cells was analysed, showing that M1 and M2-related genes were significantly upregulated in P3 and P4 patients with GSE154600 (**Figure 3D**).

### CIBERSORT

**Figure 4:**
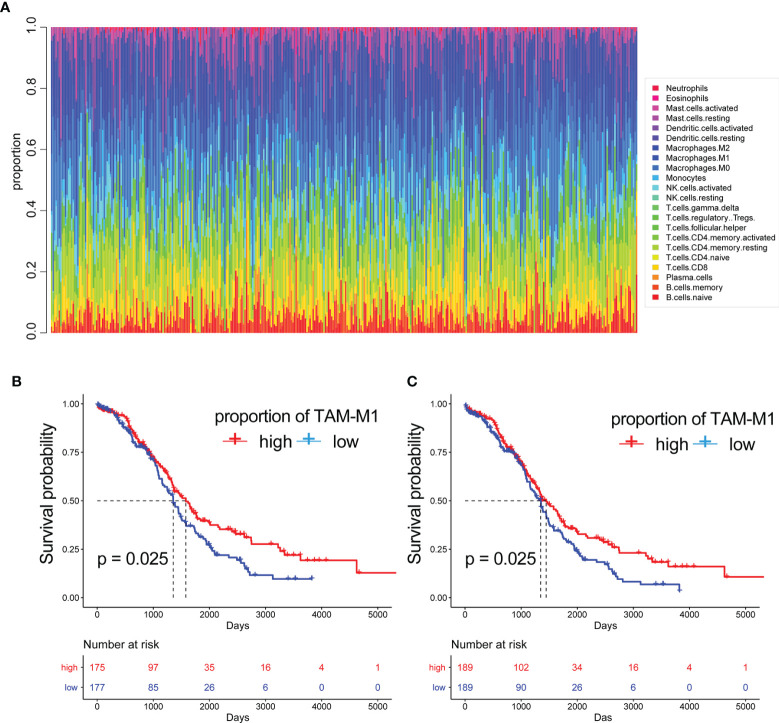
The proportion of immune cells. **(A)** The proportion of 22 immune cells built on RNA-seq count data. The Kaplan-Meier curves of patients with different proportions of M1-like myeloid cells:**(B)** are disease free interval (DFI) **(C)** overall survival (OS).

Based on the results of single-cell sequencing data and immune cell types analysis, we used bulk data for clinical significance analysis and prognostic model construction. Since bulk RNA-seq data has the advantage of more samples and more clinical information, in order to further analyze the clinical significance of immune cells infiltrated by OC. CIBERSORT can predict the proportion of 22 immune cells based on RNA-seq count data and was used to calculate the abundance of M1-like TAMs (tumour-associated macrophages) in the bulk RNA-seq data of 378 TCGA-OC patients (**Table 2**) (**Figure 4A**). The results of survival analysis showed that the patients with a high abundance of M1-TAMS had better survival (**Figures 4B, C**). There was no significant survival difference among patients with proportions of M2-like TAMS (**Supplementary Figure S1**), therefore, we conducted an in-depth analysis of M1-like TAMs.

**Table 2:** OV patient information (bulk RNA-seq).

	All dataset	Training set	Test set
Number	378	189	189
DEAD	232	116	116
Alive	146	73	73
Age > 65	132	67	65
Age <=65	238	119	119
NA	8	3	5
Stage			
Stage I	58	36	22
Stage II	23	10	13
Stage III	294	142	152
NA	3	1	2
Grade			
G1	1	0	1
G2	45	24	21
G3	321	160	161
G4	1	0	1
NA	10	5	5

### WGCNA Analysis and Immunotherapy Prediction

**Figure 5:**
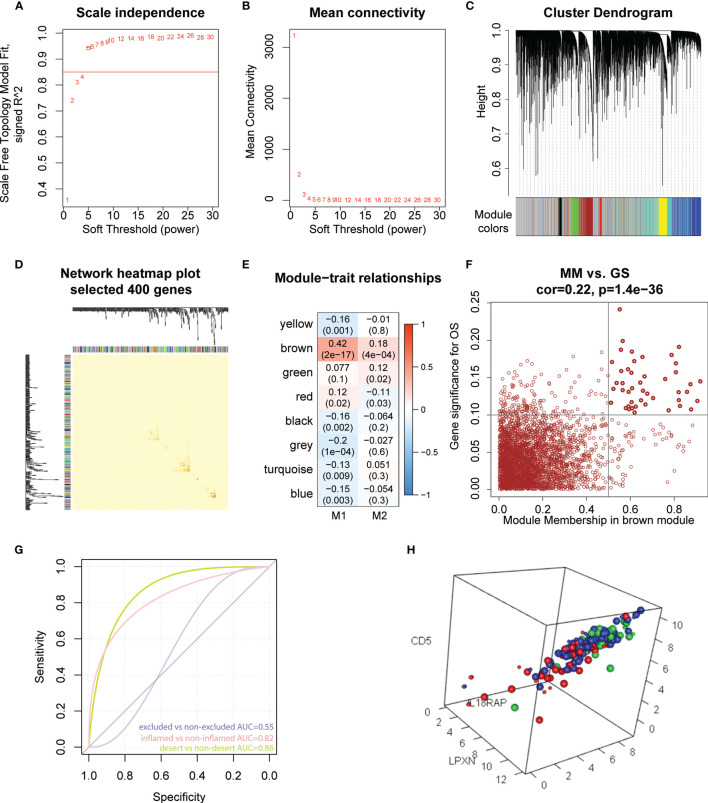
Hub genes screening and immunotherapy prediction. **(A)** The nature of the network topology constructed with unique Power values. **(B)** The relationship between Power values and average connectivity. **(C)** Genes are clustered into discrete modules. **(D)** Four hundred genes were randomly selected and clustered into distinct modules. **(E)** The correlation between different modules and the proportion of M1 and M2-like myeloid cells. **(F)** In the brown module, the correlation between genes and overall survival was reported as scatter plot, and the dark dots were hub nodes. **(G)** predicting AUC (Area Under Curve) of three phenotypes. **(H)** Different colors represented different types: Large dots represented support vector, red represented desert samples, blue represented excluded-samples, and green represented inflamed samples.

To further explore the potential role of M1-like TAMs in OC, we performed WGCNA analysis based on TCGA data (60,483 genes, 378 patients). The genes with median absolute deviation (MAD) ≤0.01 were filtered out leaving 35,165 genes. With a soft threshold =5 (**Figures 5A, B**), a scale-free co-expression network was constructed to identify gene features related to M1-like TAM. A total of 7 modules were generated (**Figures 5C, D**), of which the brown module (3213 genes) had the highest correlation with the M1-like TAM score (r=0.42, P=2e−17, **Figure 5E**). As shown in **Figure 5F**, genes are represented as points, the abscissa module membership represents the correlation between genes and module eigengene, and the ordinate represents the correlation between the gene expression and OS. The results show that the important elements of the brown module represent OS-related genes, finally obtaining 45 hub genes (MM>0.5 and GS>0.1) from the module (**Supplementary Table 4**).

IMvigor210CoreBiologies package contains RNA-seq data of 348 PD-L1 immunotherapy tumour patients classified into three phenotypes, inflamed type, immune excluded type and immune desert type. We used the e1071 package to construct a support vector machine to predict the three phenotypes (**Figure 5G**), showing that the prediction effect was better when used to distinguish between the inflamed and immune desert types (**Figure 5H**). These results indicate that 45 M1-like myeloid cell-related genes are potential predictors of immune infiltration.

### Molecular Typing Based on M1-Related Genes

**Figure 6:**
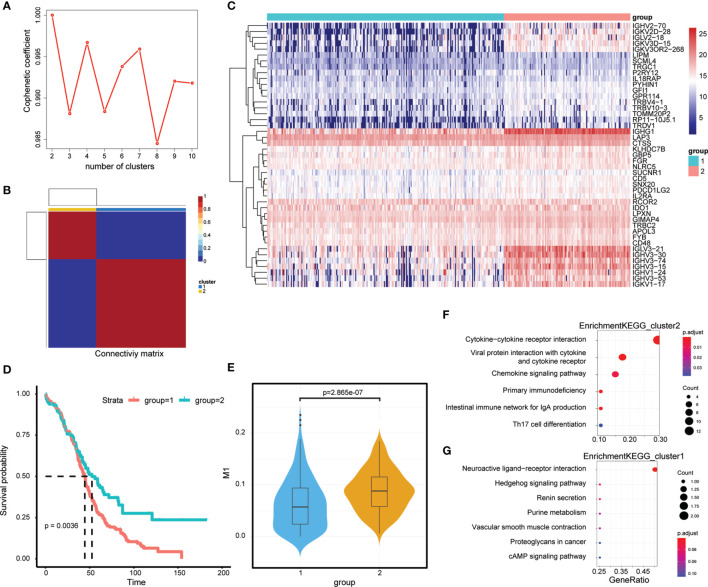
M1-related Molecular typing. **(A)** Consensus Map of NMF Clustering. **(B)** Sample cluster of TCGA-OC. **(C)** Expression of relating genes in M1 like myeloid cells. **(D)** Kaplan-Meier curves of two OC molecular subtypes. **(D)** Proportion of M1-like myeloid cells in two OC molecular subtypes. **(E)** KEGG functional enrichment analysis of differential genes in two OC molecular subtypes. **(F, G)** KEGG functional enrichment analysis of differential genes in two OC molecular subtypes.

First, the expression of 45 M1-related hub genes was extracted from the TCGA database and the NMF package was used to divide the TCGA samples into different subgroups based on non-negative matrix factorisation. The cluster = 2 as the optimal parameter (**Figure 6A**) and the training set was divided into two subgroups. Then, the consistency matrix was established (**Figure 6B**), the value of the consensus matrix is [0,1], equal to 1 means multiple clustering and two data points are all in the same class, and 0 represents that multiple clustering is not in the same class. The heat map showed the expression of 45 M1-related genes (**Figure 6C**) and the prognosis of cluster 2 is better than that of cluster 1 (**Figure 6D**). The Violin plot shows that the proportion of M1 in cluster 2 is higher than in cluster 1 (P=2.865e-07, Wilcox-test) (**Figure 6E**). In general, the prognosis of patients in cluster 1 is worse. The genes differentially expressed in cluster 2 and cluster 1 (|logFC|>2 and adj.P.val<0.05) were identified by the limma package, obtaining 658 DEGs, of which, 39 genes were downregulated (**Supplementary Table 5**) and 619 genes were upregulated in cluster 2 (**Supplementary Table 6**). The Clusterprofiler package was used to perform KEGG enrichment analysis in cluster 2 (**Figures 6F, G**).

### Construct a Genetic Risk Model

**Figure 7:**
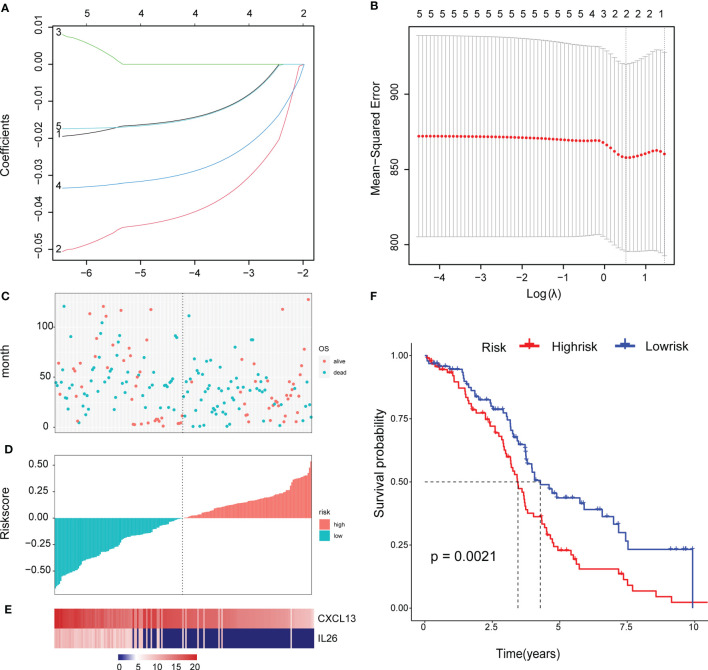
Construct a genetic risk model: **(A)** The trajectory of each independent variable: the horizontal axis represents the log value of the independent variable lambda, and the vertical axis represents the coefficient of the independent variable. **(B)** The confidence interval under each lambda. **(C)** The horizontal axis is the patient’s risk score, ranked from lowest to highest, the vertical axis is survival, the green dots are dead, and the red dots are survival. **(D)**The abscissa shows the patient’s risk score from low to high, the ordinate is the standardized risk score, the red represents the high-risk group, and the green represents the low risk group. **(E)** 2 Expression of Gene Signature in all TCGA patients. The abscissa shows the order of patients’ risk score from low to high. **(F)** Survival curve of high and low risk group.

To facilitate subsequent verification, 101 protein-coding genes were selected for subsequent analysis from the 658 DEGs (**Supplementary Table 7**). The TCGA was randomly divided into training and test sets according to a 1:1 ratio, with 189 samples in each dataset (**Table 2**). Cox (proportional hazards model) was used to identify four survival-related genes (CXCL13, PLA2G2D, IL26, CARD17) in the training set. Then, lasso regression was used to solve the multicollinearity problem during regression analysis and reduce the number of genes in the risk model. We used the glmnet package to perform lasso Cox regression analysis and the change trajectory of each independent variable is shown in the Figure. As the lambda gradually increases, the number of independent variable coefficients tends to 0 gradually increases (**Figure 7A**). Next, we used a 10-fold cross test to construct the model and confidence interval under each lambda, as shown in **Figure 7B**. The model is optimal when lambda = 0.52 and two genes (CXCL13, IL26) were chosen to construct a risk model and the prognostic KM curves of the two genes are shown in **Supplementary Figure S2**.

The final 2-gene signature is as follows: RiskScore=-0.059*CXCL13-0.034*IL26.

We calculated the risk score of the TCGA training set and determined the risk score distribution, showing that the higher the risk score and mortality rate of patients with the lower gene expression of CXCL13 and IL26 (**Figures 7C–E**). The median risk score was standardised as 0, and the samples were classified as high or low risk with median standardisation. The prognosis of the high-risk group was worse (**Figure 7F**).

### Verification of the Prognostic Risk Model

**Figure 8:**
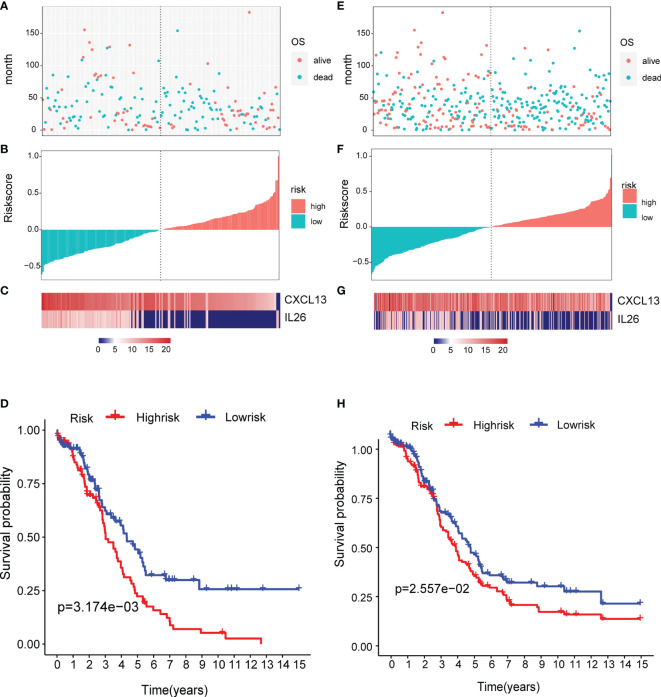
Verification of the prognostic risk model: **(A–C)** the TCGA test and **(E–G)** all TCGA datasets were used to calculate the RiskScore and distribution, showing that samples with a high RiskScore were significantly smaller than those with a low RiskScore. **(D, H)** The results of the KM curves shown in reveal significant differences between the low and high-risk group.

To determine the robustness of the model, we used the TCGA test (**Figures 8A–C**) and all TCGA datasets (**Figures 8E–G**) to calculate the RiskScore and distribution, showing that samples with a high RiskScore were significantly smaller than those with a low RiskScore. Low expression of CXCL13 and IL26 was identified as a risk factor. Finally, the results of the KM curves shown in **Figures 8D, H** reveal significant differences between the low and high-risk group (p < 0.05).

### The Expression of Signature Genes in OC Tissues

**Figure 9:**
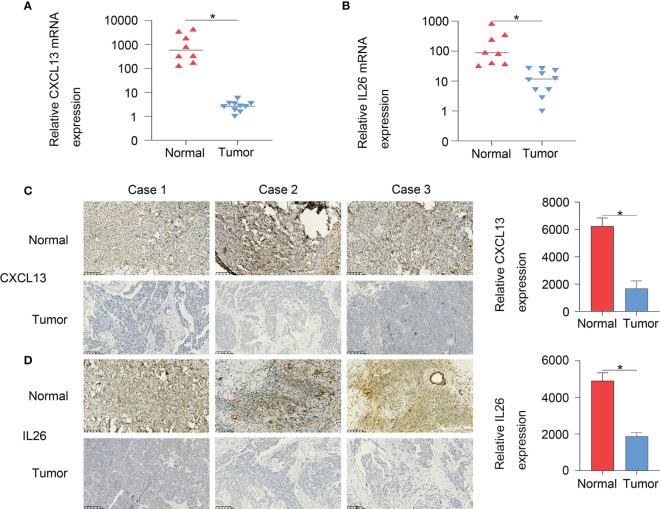
The qPCR **(A, B)** and IHC **(C, D)** results showed that CXCL13 and IL26 expression were low in OC tissues, *p < 0.05.

Furthermore, to verify the accuracy of the two-gene signature, we examined the expression of the signature genes (CXCL13 and IL26) in clinical samples from OC patients by qPCR (**Figures 9A, B**) and IHC (**Figures 9C, D**) analysis, showing that the expression of CXCL13 and IL26 was low in OC tissues.

## Discussion

Although PD-L1/PD-1 as targets for immunotherapy have been identified and the prognosis of most immunotherapy cancer patients has been effectively improved, such as lung cancer (10), breast cancer (11) and haematological tumours (12), immunotherapy for OC is not very effective (13). There is mounting evidence to suggest that intratumoral heterogeneity exists in cells within OC, which makes it rather challenging to identify effective immunotherapeutic targets. The current research shows that single-cell genomics is a powerful tool to explore tumour heterogeneity and distinct subpopulations, which is important to identify potential therapeutic targets (14–17).

In this study, two scRNA-seq datasets (GSE154600 and GES158937) were used to characterise the OC heterogeneity. Normalisation and variance stabilisation of the two scRNA-seq datasets using regularised negative binomial regression by SCTransform () revealed 20 clusters. According to immune cell markers, T or NK cells (cluster 5, 6, 7, 11; markers: CD3D and CD3E), B cells (cluster 16, 19, 20; marker: CD79A) and myeloid cells (cluster 4, 9, 10; LYZ and CD14) were clustered. Then, we identified immune-related OC cells based on the GSVA enrichment score of each sample of cells, showing that tumour-infiltrated myeloid cells and the activity of M2 and M1-like myeloid cells were significantly upregulated in P3 and P4 patients with GSE154600 data. Here, we explored the intratumoral heterogeneity by analysis of the two OC scRNA-seq datasets and the differential interactions between tumour and myeloid cells based on immune cell subtype. Next, TCGA-OC bulk RNA-seq data (including 378 ovarian cancer patients and 58385) were used for predicting the proportion of 22 immune cells and calculating the abundance of M1-like TAMs. The survival analysis showed that the patients with a high abundance of M1-TAMS had better survival, in line with recent findings of the involvement of innate immunosuppression driven by myeloid-derived suppressor cells in the development of ovarian cancer (18–20). Furthermore, we first revealed that tumour-associated macrophages, such as M1-TAMS, are closely related to survival and there was no significant survival difference among patients with proportions of M2-like TAMS. We further explored the potential role of M1-like TAMs in OC and performed WGCNA analysis based on TCGA-OC data, showing that the important elements of the brown module represent OS-related genes. Finally, 45 hub genes were obtained from the module. Based on the M1-related genes, the TCGA-OC training set was divided into two different subgroups (cluster 1 and cluster 2), and Cox was used to identify four survival-related genes (CXCL13, PLA2G2D, IL26, CARD17). The two-gene signature was RiskScore=-0.059*CXCL13-0.034*IL26 based on lasso Cox regression analysis. To verify the prognostic risk model, we used the TCGA test and all TCGA datasets to calculate the RiskScore and distribution, showing that low expression of CXCL13 and IL26 was a risk factor. Furthermore, to verify the accuracy of the two-gene signature, qPCR and IHC analysis revealed that the expression of CXCL13 and IL26 was low in OC tissues, demonstrating that the two-gene signature provides valuable resources to accurately evaluate the prognostic risk.

Recently, there has been increasing evidence to suggest that CXCL13 and IL26 could be potential targets for OC. It has been suggested that the CXCL13 may play a crucial role in the development, metastasis and relapse of advanced colon cancer, and can be used as a prognostic marker for colon cancer (21). For clear cell renal cell carcinoma, gastric cancer, breast cancer and hepatocellular carcinoma (22–25), CXCL13 had good diagnostic and prognostic value, hence may become a candidate biomarker and therapeutic target. Many other investigators have demonstrated the promising role of IL26 with immunotherapy in treating cancers. For example, IL26 is a unique, clinically relevant, inflammatory amplifier that enhances TNBC (triple negative breast cancer) engraftment and dissemination in association with neutrophils, which has the potential as a therapeutic target (26). The serum IL-26 level is closely correlated with gastric cancer and has important value for the determination of disease occurrence and development (27). Yang Moran et al. revealed that CXCL13 can shape the antitumor microenvironment and support a clinical investigation for a combination of CXCL13 and PD-1 blockade therapy in HGSC (28). Winkler et al. attempted to introduce new therapies based on Th17 lymphocytes which produce IL-17A, IL-17F, IL-21, IL-22, IL-26, IL-6, TNF-α and suppress tumour progression through enhanced antitumor immunity in OC (29). For the first time, we proposed a two-gene signature (CXCL13 and IL26) based on the heterogeneity of OC, which may be applied for risk prediction and as potential immunotherapy targets. However, this study has some limitations, such as few samples for PCR and immunohistochemical verification and the mechanism of CXCL13 and IL26 has not been explored in OC. Future efforts should focus on using many samples to verify the accuracy of the model and explore the molecular mechanism of CXCL13 and IL26, providing experimental evidence for application to risk prediction and treatment in OC.

In conclusion, two scRNA-seq datasets (GSE154600 and GES158937) were integrated and used to characterise OC heterogeneity, with the M1 and M2-related genes significantly upregulated in P3 and P4 patients with GSE154600. Our work not only expands the understanding of tumour-infiltrated myeloid cells but also provides a two-gene signature based on M1-related genes in the TCGA-OC data. The combined analysis of single-cell data and TCGA-OC data identified the two-gene signature with important prognostic implications and immunotherapy in OC.

## Data Availability Statement

The original contributions presented in the study are included in the article/**Supplementary Material**. Further inquiries can be directed to the corresponding author.

## Ethics Statement

The studies involving human participants were reviewed and approved by CAMS & PUMC (17-099/1355). The patients/participants provided their written informed consent to participate in this study.

## Author Contributions

LW and NL conceived and designed the study. JY, LL, and JLi performed the experiments. JLiu, JZ, and TW analyzed the data and drafted the manuscript. All authors contributed to the article and approved the submitted version.

## Funding

This work was supported by National Key R&D Program of China (2016 YFC1303700).

## Conflict of Interest

The authors declare that the research was conducted in the absence of any commercial or financial relationships that could be construed as a potential conflict of interest.

## Publisher’s Note

All claims expressed in this article are solely those of the authors and do not necessarily represent those of their affiliated organizations, or those of the publisher, the editors and the reviewers. Any product that may be evaluated in this article, or claim that may be made by its manufacturer, is not guaranteed or endorsed by the publisher.

## Supplementary Material

Supplementary Figure S1 The Kaplan-Meier curves of patients with different proportions of M1-like myeloid cells: **(B)** disease free interval(DFI) **(C)** overall survival(OS).
