# Supplementary figures and images for: Integration of scRNA-Seq and Bulk RNA-Seq to Analyse the Heterogeneity of Ovarian Cancer Immune Cells and Establish a Molecular Risk Model
Source: Front Oncol. 2021 Sep 21;11:711020. doi: 10.3389/fonc.2021.711020 (PMC8490743; doi:10.3389/fonc.2021.711020)

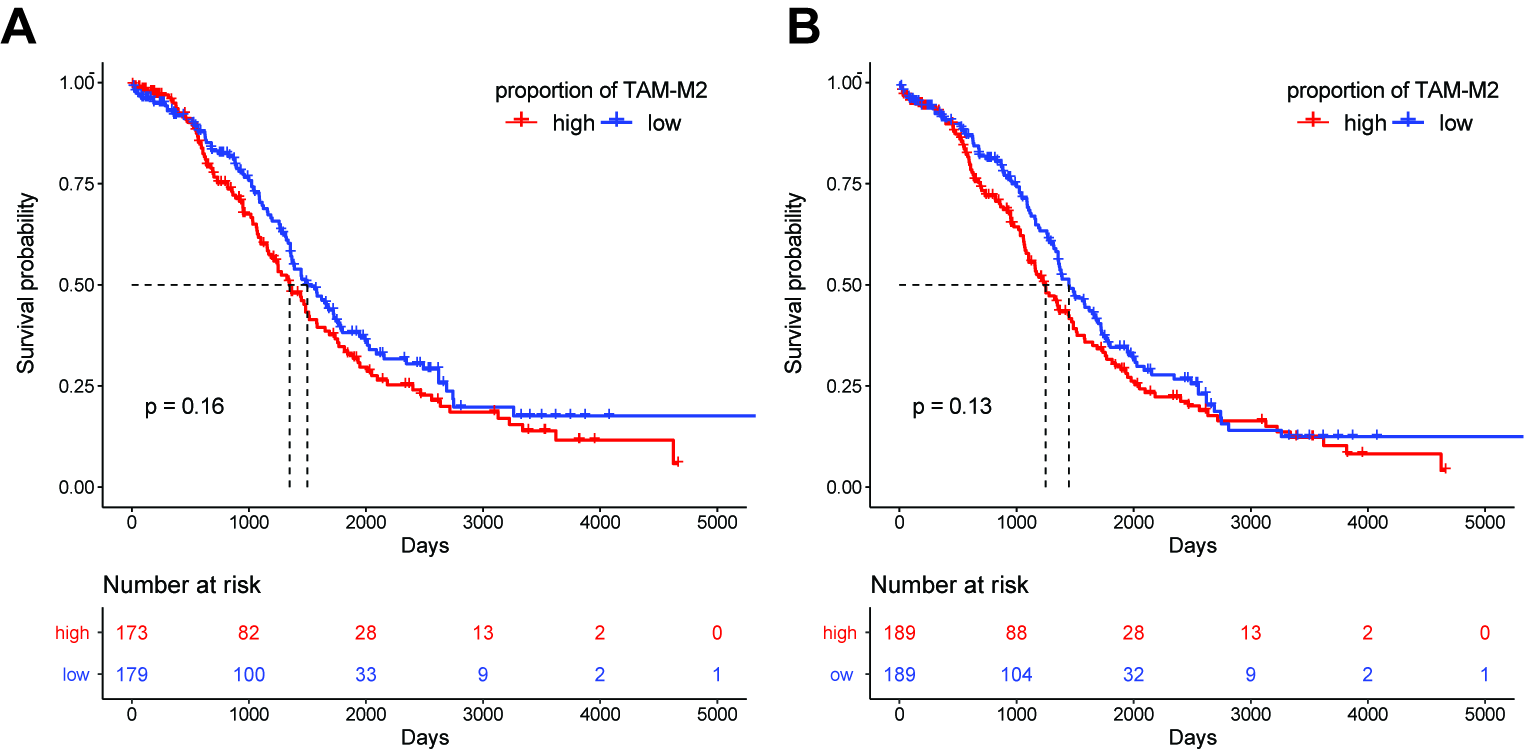

Supplement: Supplementary Figure S1 — The Kaplan-Meier curves of patients with different proportions of M1-like myeloid cells: (B) disease free interval(DFI) (C) overall survival(OS). [file Image_1.tif]

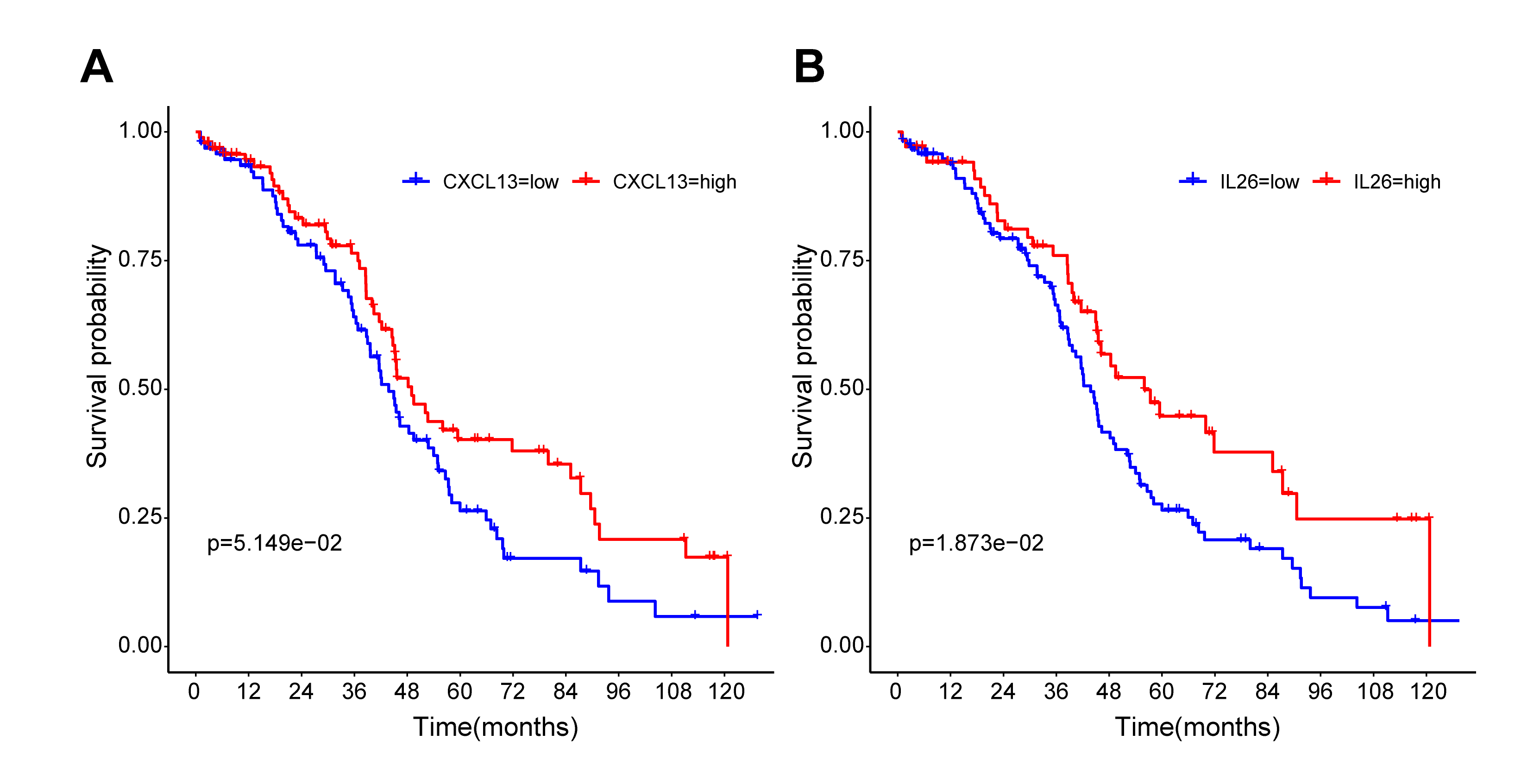

Supplement: Supplementary file 2 [file Image_2.tif]
